# Atrial fibrillation detection in primary care during blood pressure measurements and using a smartphone cardiac monitor

**DOI:** 10.1038/s41598-021-97475-1

**Published:** 2021-09-06

**Authors:** John D. Sluyter, Robert Scragg, Malakai ‘Ofanoa, Ralph A. H. Stewart

**Affiliations:** 1 School of Population Health, University of Auckland, Private Bag 92019, Auckland, New Zealand; 2 Green Lane Cardiovascular Service, Auckland City Hospital, Auckland, New Zealand

**Keywords:** Cardiology, Health care

## Abstract

Improved atrial fibrillation (AF) screening methods are required. We detected AF with pulse rate variability (PRV) parameters using a blood pressure device (BP+; Uscom, Sydney, Australia) and with a Kardia Mobile Cardiac Monitor (KMCM; AliveCor, Mountain View, CA). In 421 primary care patients (mean (range) age: 72 (31–99) years), we diagnosed AF (n = 133) from 12-lead electrocardiogram recordings, and performed PRV and KMCM measurements. PRV parameters detected AF with area under curve (AUC) values of up to 0.92. Using the mean of two sequential readings increased AUC to up to 0.94 and improved positive predictive value at a given sensitivity (by up to 18%). The KMCM detected AF with 83% sensitivity and 68% specificity. 89 KMCM recordings were “unclassified” or blank, and PRV detected AF in these with AUC values of up to 0.88. When non-AF arrhythmias (n = 56) were excluded, the KMCM device had increased specificity (73%) and PRV had higher discrimination performance (maximum AUC = 0.96). In decision curve analysis, all PRV parameters consistently achieved a positive net benefit across the range of clinical thresholds. In primary care, AF can be detected by PRV accurately and by KMCM, especially in the absence of non-AF arrhythmias or when combinations of measurements are used.

## Introduction

Atrial fibrillation (AF) is the most common cardiac arrhythmia and is associated with a significantly increased risk of stroke^1^. Stroke risk can be significantly diminished with effective anticoagulation for those deemed to have a sufficiently high risk according to risk scores (e.g., CHA_2_DS_2_-VASc), making early AF detection important^2^. However, AF is often diagnosed not early, but after a stroke event^3^. Thus, new methods to detect AF sooner are needed.

AF can be detected opportunistically during automatic blood pressure (BP) measurement by assessing pulse rate variability (PRV) from the beat-to-beat timing (irregularity of duration) of BP waveforms; useful since BP is routinely assessed. Diagnostic studies show that this approach detects AF accurately with high sensitivity and specificity^4,5^; more so than other non-ECG AF screening devices^6^. These studies have been almost exclusively carried out in outpatients. However, the applicability of these findings to primary care patients, who are most likely to benefit from opportunistic screening, may be uncertain as the performance of screening tests varies between populations and clinical settings due to the spectrum effect that can arise when results from a hospital study are applied in the community setting of general practice^7^. QUAD-2 tool appraisal (quality assessment) of these studies has highlighted other shortcomings, including that the statistical analyses in some studies treated repeated measurements as separate observations, which is likely to break the assumption that they are independent by being correlated^5^. Further, as there was only one threshold of the PRV classifier evaluated for discrimination performance in each study (not a range), the study findings do not tailor to a range of clinical preferences to balance false-positives and false-negatives^8^.

Another AF screening instrument is the Kardia Mobile Cardiac Monitor (KMCM; AliveCor, Mountain View, CA): a hand-held, smartphone-coupled, 2-electrode cardiac rhythm recorder that generates a rhythm strip equivalent to lead I for 30 s. Diagnostic studies show the KMCM detects AF accurately with high sensitivity and specificity (both > 90%)^9–11^. However, it is not always (e.g., 28% of the time^12^) able to generate interpretable recordings and give “normal” or “possible AF” classifications of these—and this results in missed cases and reduced screening performance (e.g., sensitivity = 77%, specificity = 76%)^12,13^. For unclassified readings, reliance on manual interpretation is not suitable if performed by inadequately skilled clinical staff^14^ and, if interpreted by cardiologists^13^, requires consideration of their busy workloads. One way to address this problem could be to use KMCM in combination with AF detection from a BP monitor as the latter would give a classification when KMCM does not. But this approach has not been previously investigated.

Given the knowledge gap, we evaluated how well PRV measured using a BP monitor (*BP*+ *device*; Uscom, Sydney, Australia) screens for AF in primary care. We hypothesised that this could detect AF accurately^15^, thereby making it an attractive screening method as PRV can be assessed during measurement of both BP and other parameters that predict cardiovascular events^16–18^. To build on past research, we assessed screening performance across a range of PRV thresholds for AF detection by this instrument. Second, we examined the AF screening performance of KMCM, including when used together with PRV.

## Methods

### Participants

Participants were recruited from four primary care practices in Auckland. Inclusion criteria were men and women from these clinics. Participants were identified electronically from the patient database by practice staff. To help ensure an adequate number of AF cases in our sample, we selected patients with a prior AF diagnosis. To recruit those without AF, we selected patients without a prior AF diagnosis. For this selection we used the database to calculate 5-year AF risk^19^ and recruited from highest to lowest risk in order to further increase the chances of recruiting sufficient AF cases. An information sheet and personalised invitation letter (from the clinic) were mailed to each home. This was followed up by clinic staff contacting each patient by phone to schedule an interview time at the practice (for those interested). Nearly all patients were recruited this way and the remaining patients were recruited opportunistically: during routine consultations, eligible patients deemed as having a high AF risk were invited to participate. Ethics approval was provided by the Ministry of Health Central Health and Disability Ethics committee. Written, informed consent was obtained from each participant. All methods were carried out in accordance with the relevant guidelines and regulations.

### Measurements

All measurements were performed by trained clinic staff using a standardised protocol. Demographic and past medical history data were collected via questionnaires administered by staff. Past medical history was also captured from the patient management system at each clinic.

After > 15 min rest and in the semi-reclining position, a 12-electrode lead ECG was performed to assess heart rhythm. To diagnose AF cases and controls, the ECG readings was read by a senior (consultant) cardiologist (R.A.H.S.), who was blinded to the corresponding PRV data (described next). Each ECG report also noted if other arrhythmias were present.

Simultaneously to the ECG measurements or up to 1 min after this, suprasystolic oscillometry was carried out twice using a BP+ device (Uscom, Sydney, Australia), with an appropriately-sized cuff positioned over the upper arm. Results presented are based on the first measurement only, unless otherwise indicated. PRV was assessed from the variability of the beat duration of the suprasystolic pressure waveforms derived from the BP+ device using custom-written Matlab software (Mathworks, Natick, MA)^20^. The waveforms spanned approximately 10 s; thus analysis was performed on approximately 10–12 pulse intervals. Six measures were used: (1) standardised average real variability (standardised ARV; sARV), the mean of the absolute pulse-interval differences across consecutive beats (analogous to ARV in BP variability measurement^17^), as a percentage of the mean of these intervals, (2) root mean square of successive differences (RMSSD, square root of the mean of the squared differences of the duration of successive pulse intervals)^15,18^, (3) standard deviation (SD)^21–23^, (4) coefficient of variation (CV; SD as a percentage of the mean pulse interval; reported as irregularity index in previous studies^15,18,22,23^), (5) relative range, the range (maximum minus minimum) of all pulse intervals across beats divided by their mean (comparable to relative range in BP variability assessment^17^) and, (6) irregular pulse percentage (IPP), the number of irregular pulses as a percentage of the total number of pulses^24^. For IPP, pulses were defined as irregular if they were >  ± 15%^24^ the duration of the mean beat. For the remaining parameters, to reduce false positives (e.g., ectopic beats), we excluded beats that were 25%^21–23^ to 30% shorter or longer than the mean beat duration. These percentage differences were chosen as they provided optimal discrimination performance in our dataset (Supplementary Fig. 1). Representative pulse recordings from the BP+ device are illustrated in Supplementary Fig. 2.

A KMCM, described above, was used immediately after to record a 30-s rhythm strip. The algorithm it uses gives “normal” or “possible atrial fibrillation” classifications to recordings. If it cannot classify a recording to a sufficient degree of confidence or if heart rate is < 50 or > 100 beats/minute and regular, the recording is labelled “unclassified”^12^.

### Statistical analysis

Analyses for participant characteristics were carried out using SAS version 9.4 (SAS Institute, Cary, NC) and all others were performed using R version 3.6.3^25^. Receiver operating characteristics (ROC) curves were constructed to determine the ability of PRV parameters to correctly classify individuals on the basis of their ECG-diagnosis result (AF present or absent). We assessed discrimination performance with area under the ROC curve (AUC; with 95% confidence intervals) for PRV and with diagnostic accuracy for both devices. These values represent the proportion of individuals that are correctly classified. Further, we quantified sensitivity, specificity, positive predictive value (PPV), negative predictive value and F1 score. F1 score is a weighted average of sensitivity and PPV ranging from 0 to 1, with a high score indicating low false positive and negatives^26^. To more stringently evaluate the validity of the PRV parameters, we performed a tenfold cross-validation 10 times.

We performed multiple subgroup analyses. First, we excluded people with a non-AF arrhythmia as this could reduce specificity^15^. Second, we omitted individuals with a rhythm that was paced as this could be regular in those with AF (reducing sensitivity). Third, for PRV, we excluded those with a low pulse rate (defined as < 48 beats/minute) as the associated relatively few number of beats (< 8 over the ~ 10-s recording period) could potentially screen for AF less accurately. Fourth, we performed a subgroup analysis (for PRV) that was limited to those with unclassified/blank KMCM readings and another (for the KMCM) that excluded these readings. Given the importance of detecting new AF cases, we performed a fifth subgroup analysis in those without a prior AF diagnosis. Sixth, we restricted analysis to participants aged ≥ 65 years as clinical guidelines suggest opportunistic screening in this age group^4^. As screening based on multivariable risk models has been suggested as an alternative to age, we performed a seventh subgroup analysis in those with 5-year AF risk ≥ 5%^19^. Finally, anticoagulant therapy (for stroke prevention) has been recommended for AF patients with a CHADS_2_ score of ≥ 2 or with a CHA_2_DS_2_-VASc score of ≥ 1 (in males) or ≥ 2 (in females)^2^. As those with these elevated stroke risk scores would benefit most from AF screening^22^, we performed subgroup analyses in these individuals.

For the PRV parameters, we calculated net benefit from decision curve analysis. The decision curve analysis incorporates the information on both the benefit of correctly predicting the outcome (true-positives) and the relative harm of over-reporting it (false-positives)—that is, the net benefit^8^. For instance, after putting these benefits and harms on the same scale (by adjusting for their relative value), a net benefit of 0.1 means that, per 100 patients, there are 10 more benefits than harms^8^. As decision curves, we made a graphical presentation of the net benefit over a range of threshold probabilities (or clinical preferences) of the outcome—for each PRV parameter, for the default assumption that none have AF (not testing anyone for this outcome) and for the assumption that all do (testing everyone for AF). Precision-recall curves were constructed to illustrate the relationship between PPV (precision) and sensitivity (recall)^27^. To summarise precision across sensitivities, we quantified area under these curves (range: 0 to 1), with higher area indicating better overall precision^27^. We used more than one screening measurement to indicate AF as this can increase confidence in predictions^4^. We constructed a second set of curves based on PPV at an AF prevalence of 10%^28^ to assess performance in a less targeted population^18^.

## Results

There were 421 participants (56% male), 133 of whom were diagnosed as having AF based on their 12-lead ECG at their study visit. Age was 72 years on average and ranged from 31 to 99 years. Nearly three-quarters (n = 309) were of Pacific ethnicity. These characteristics and others are summarised in Table 1.

**Table 1:** Characteristics of participants.

Variable	12-ECG diagnosis from study visit	
	AF absent	AF present
**n**	288 (100)	133 (100)
**Age (years)**	72 ± 12	71 ± 11
**Male sex**	151 (52)	84 (63)
**Ethnicity**		
Pacific	242 (84)	66 (50)
Maori	5 (2)	11 (8)
Asian	5 (2)	2 (2)
European/other	36 (13)	54 (41)
No prior AF diagnosis	208 (72)	9 (7)
**12-ECG diagnosis from study visit**		
Premature atrial contraction	14 (5)	0 (0)
Premature ventricular contraction	15 (5)	11 (8)
Supraventricular rhythm	1 (0.4)	0 (0)
Supraventricular tachycardia	1 (0.4)	0 (0)
Atrial arrhythmia	1 (0.4)	0 (0)
AV block (2nd-degree or complete)	1 (0.4)	0 (0)
Pacemaker	6 (2)	7 (5)
Junctional rhythm	3 (1)	1 (1)
**12-lead ECG heart rate (beats/min)**	70 ± 15	76 ± 15
**PRV parameters**		
Standardised ARV (%)^†^	2.2 ± 2.9	16.2 ± 7.4
RMSSD (ms)^†^	23 ± 34	139 ± 104
Standard deviation (ms)^†^	18 ± 28	112 ± 67
Coefficient of variation (%)^†^	2.1 ± 2.7	12.6 ± 5.7
Relative range (%)^†^	6.6 ± 10.2	43.2 ± 16.7
Irregular pulse period (%)^†^	9.1 ± 11.0	50.0 ± 35.0
Pulse rate > 48 beats/minute	277 (96)	125 (94)
**KMCM diagnosis**		
Normal	195 (68)	3 (2)
Possible AF	23 (8)	111 (83)
Unclassified	57 (20)	15 (11)
Blank (no analysis)	13 (5)	4 (3)
5-year AF risk ≥ 5%	185 (64)	71 (53)
High CHADS_2_ score (≥ 2)	219 (76)	84 (63)
High CHA_2_DS_2_-VASc score^‡^	274 (95)	130 (98)

*AF* atrial fibrillation, *AV* atrioventricular, *KMCM* Kardia Mobile Cardiac Monitor, *PRV* pulse rate variability, *RMSSD* root mean square of successive differences. *Values are n (column %) or mean ± standard deviation, unless otherwise indicated; ^†^Median ± interquartile range; ^‡^CHA_2_DS_2_-VASc score ≥ 1 (males) or ≥ 2 (females).

### PRV for detecting AF

**Figure 1:**
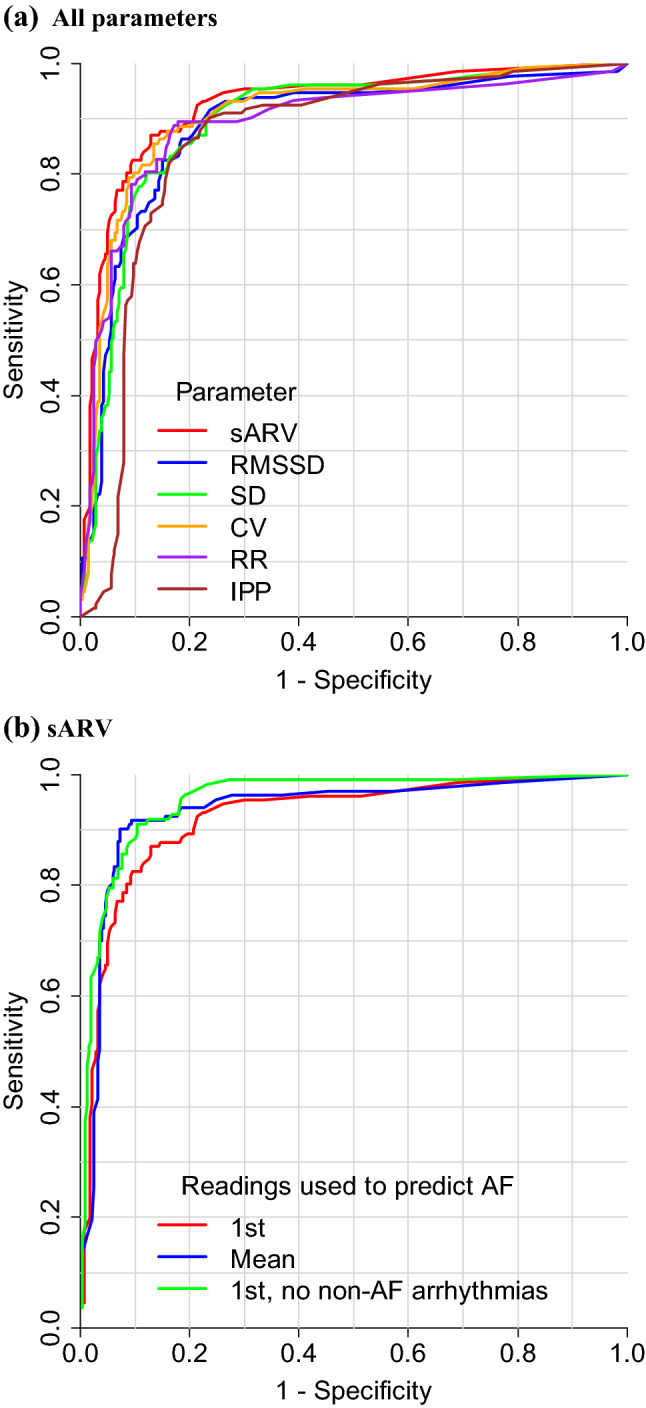
Receiver operating characteristics curves for AF detection from pulse rate variability by: (**a**) all parameters and, (**b**) sARV. *1st* 1st reading, *CV* coefficient of variation, *IPP* irregular pulse percentage, *RMSSD* root mean square of successive differences, *RR* relative range, *sARV* standardised average real variability, *SD* standard deviation.

All PRV parameters were substantially higher in people with AF than in those without (Table 1). The discrimination performance of PRV in detecting AF is illustrated in Fig. 1 and summarised in Table 2, with threshold-specific performances provided in Supplementary Tables 1–3. In the total sample, sARV had the highest AUC (of 0.92) and IPP had the lowest (AUC = 0.86). Using the mean of the first and second PRV readings (the median time between these was 95 s) for detection instead yielded higher AUC values (by up to 0.04). These parameters also performed well in the cross-validation analysis, with AUC values of 0.89–0.92. When we excluded people with a non-AF arrhythmia (n = 56), AUC increased by 0.02–0.05 to up to 0.96 (for sARV). At high thresholds, this was due to improvements in mostly specificity as this increased more than sensitivity at a given cut-point (Supplementary Tables 1 and 3). AUC was slightly higher (by up to 0.02) in individuals without a pacemaker (n = 408). Exclusion of those with a low pulse rate (< 48 beats/minute; n = 19) did not alter discrimination performance compared to in the total sample. When analysis was restricted to those with unclassified/blank KMCM readings (n = 89), AUC values remained high (up to 0.88). Similarly, discrimination performance was high in those without a prior AF diagnosis (AUC = 0.87–0.95), and in those aged ≥ 65 years or with elevated 5-year AF risk, CHADS_2_ and CHA_2_DS_2_-VASc scores (AUC = 0.84–0.92).

**Table 2:** Discrimination performance of pulse rate variability parameters for detecting AF.

Sample	Area under curve (95% confidence interval)					
	sARV	RMSSD	SD	CV	Relative range	IPP
**Total (n = 421)**						
1st measurement	0.92 (0.89–0.95)	0.89 (0.85–0.92)	0.89 (0.86–0.93)	0.91 (0.87–0.94)	0.89 (0.85–0.93)	0.86 (0.82–0.90)
2nd measurement	0.93 (0.90–0.95)	0.90 (0.87–0.93)	0.92 (0.88–0.95)	0.92 (0.88–0.95)	0.91 (0.87–0.95)	0.85 (0.81–0.89)
Mean of 2 measurements	0.94 (0.91–0.96)	0.92 (0.89–0.95)	0.92 (0.88–0.95)	0.93 (0.90–0.96)	0.93 (0.90–0.97)	0.87 (0.84–0.91)
Repeated* tenfold cross-validation	0.89 (0.79–0.97)	0.92 (0.87–0.99)	0.89 (0.79–0.97)	0.90 (0.77–0.98)	0.91 (0.80–0.99)	0.90 (0.78–0.98)
**Subgroups**						
Without non-AF arrhythmias (n = 365)	0.96 (0.93–0.98)	0.91 (0.88–0.95)	0.93 (0.90–0.96)	0.95 (0.92–0.97)	0.92 (0.89–0.96)	0.91 (0.87–0.94)
Without paced rhythms (n = 408)	0.94 (0.92–0.97)	0.90 (0.87–0.94)	0.91 (0.88–0.94)	0.92 (0.89–0.95)	0.91 (0.87–0.94)	0.87 (0.84–0.91)
Pulse rate > 48 beats/min (n = 402)	0.92 (0.89–0.95)	0.89 (0.85–0.93)	0.90 (0.87–0.94)	0.91 (0.87–0.94)	0.89 (0.85–0.93)	0.88 (0.84–0.91)
Unclassified/blank KMCM readings (n = 89)	0.86 (0.77–0.95)	0.88 (0.81–0.96)	0.86 (0.77–0.95)	0.85 (0.76–0.95)	0.82 (0.71–0.93)	0.76 (0.64–0.89)
Without prior AF diagnosis (n = 217)	0.95 (0.91–1.00)	0.95 (0.91–0.99)	0.92 (0.87–0.98)	0.92 (0.85–0.99)	0.92 (0.84–1.00)	0.87 (0.80–0.95)
Age ≥ 65 years (n = 314)	0.91 (0.88–0.95)	0.87 (0.82–0.92)	0.87 (0.82–0.92)	0.89 (0.85–0.93)	0.88 (0.83–0.93)	0.86 (0.82–0.91)
5-year AF risk ≥ 5% (n = 256)	0.89 (0.84–0.94)	0.84 (0.78–0.90)	0.86 (0.81–0.92)	0.86 (0.81–0.92)	0.84 (0.78–0.91)	0.86 (0.81–0.91)
High CHADS_2_ score^†^ (n = 303)	0.91 (0.87–0.95)	0.87 (0.83–0.92)	0.88 (0.84–0.92)	0.89 (0.84–0.93)	0.88 (0.83–0.93)	0.85 (0.80–0.89)
High CHA_2_DS_2_-VASc score^‡^ (n = 404)	0.92 (0.89–0.95)	0.89 (0.85–0.93)	0.90 (0.86–0.93)	0.91 (0.87–0.94)	0.89 (0.85–0.93)	0.86 (0.82–0.90)

*AF* atrial fibrillation, *CV* coefficient of variation, *IPP* irregular pulse percentage, *KMCM* Kardia Mobile Cardiac Monitor, *RMSSD* root mean square of successive differences, *sARV* standardised average real variability, *SD* standard deviation. *10 times. ^†^CHADS_2_ score ≥ 2; ^‡^CHA_2_DS_2_-VASc score ≥ 1 (males) or ≥ 2 (females).

**Figure 2:**
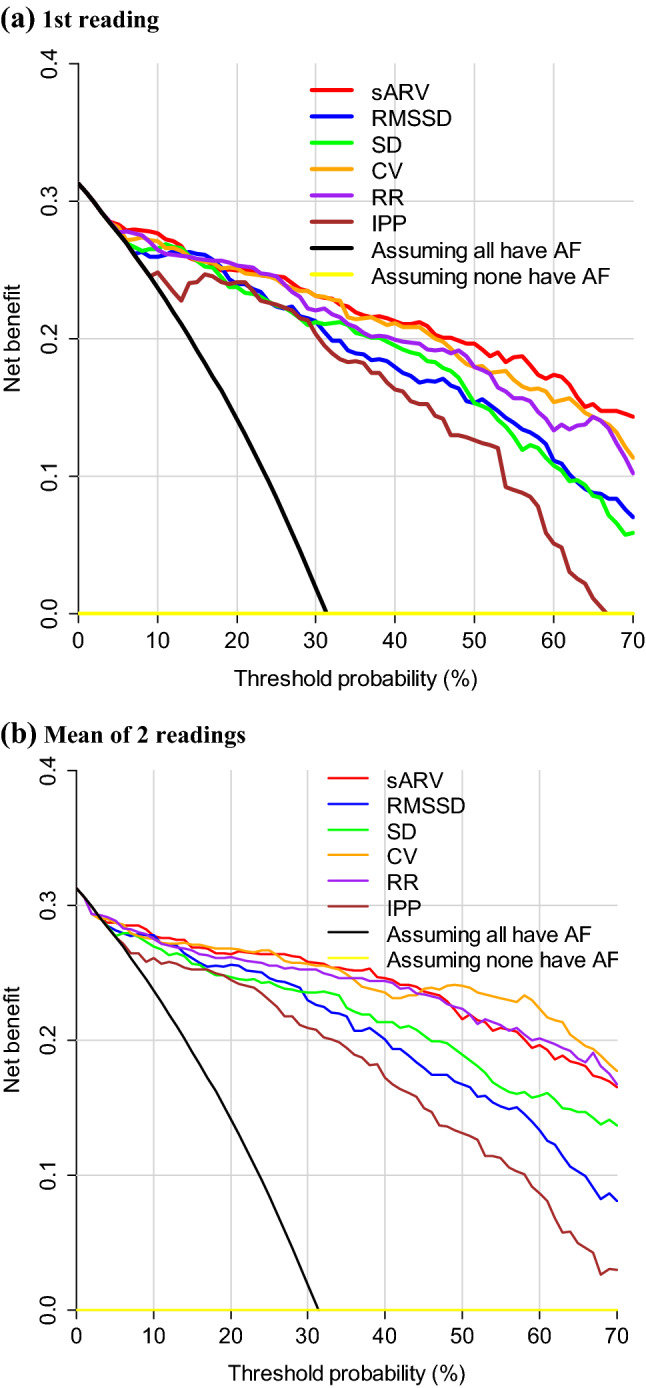
Decision curves for AF detection by pulse rate variability parameters with the: (**a**) 1st reading and, (**b**) mean of 2 readings. Abbreviations are as for Fig. 1.

As demonstrated by decision curve analysis (Fig. 2), compared to the assumptions that none or all had AF, all measures consistently had higher net benefit over the range of threshold probabilities. Net benefit for the first reading was highest overall with sARV (Fig. 2a). In comparison, net benefit was higher when the mean of two readings were used (e.g., exceeding 0.2 over a wider range of thresholds; Fig. 2b).

### KMCM for detecting AF

Table 3 shows the diagnostic performance of the KMCM for detecting AF, with supporting information (classification frequencies) provided in Table 1. Of the 421 KMCM recordings, 198 were classified as “normal”, 134 “possible atrial fibrillation” and 72 “unclassified”. In the remaining 17 recordings, the ECG output was blank as a reading could not be obtained despite multiple attempts. On assessing the accuracy of all recordings, with “unclassified” and blank recordings (both deemed as incorrect) included, the KMCM had 83% sensitivity, 68% specificity and a diagnostic accuracy of 73%. When the “unclassified” and blank recordings were excluded from the analysis, there were increases in sensitivity (to 97%), specificity (to 89%) and diagnostic accuracy (to 92%). Overall, the KMCM did not detect 17% of the 133 ECG-determined AF cases; 68% of these were due to “unclassified” KMCM recordings. Excluding participants with a non-AF arrhythmia increased sensitivity and specificity to 86% and 73%, respectively. Sensitivity was higher (by 3%) in individuals without a pacemaker. Restricting analysis to those with high CHADS_2_ and CHA_2_DS_2_-VASc scores had little effect on diagnostic accuracy (71% and 73%, respectively).

**Table 3:** Diagnostic performance of the KMCM for detecting AF.

Sample	Sensitivity	Specificity	Diagnostic accuracy (%)
Total (n = 421)	83	68	73
**Subgroups**			
Unclassified/blank readings excluded (n = 332)	97	89	92
Non-AF arrhythmias excluded (n = 365)	86	73	77
Paced rhythms excluded (n = 408)	86	68	74
No prior AF diagnosis (n = 217)	67	67	67
Age ≥ 65 years (n = 314)	83	68	72
5-year AF risk ≥ 5% (n = 256)	79	64	68
High CHADS_2_ score* (n = 303)	81	67	71
High CHA_2_DS_2_-VASc score^†^ (n = 404)	84	68	73

*AF* atrial fibrillation, *KMCM* Kardia Mobile Cardiac Monitor.

*CHADS_2_ score ≥ 2; ^†^CHA_2_DS_2_-VASc score ≥ 1 (males) or ≥ 2 (females).

### Positive-prediction sample

**Figure 3:**
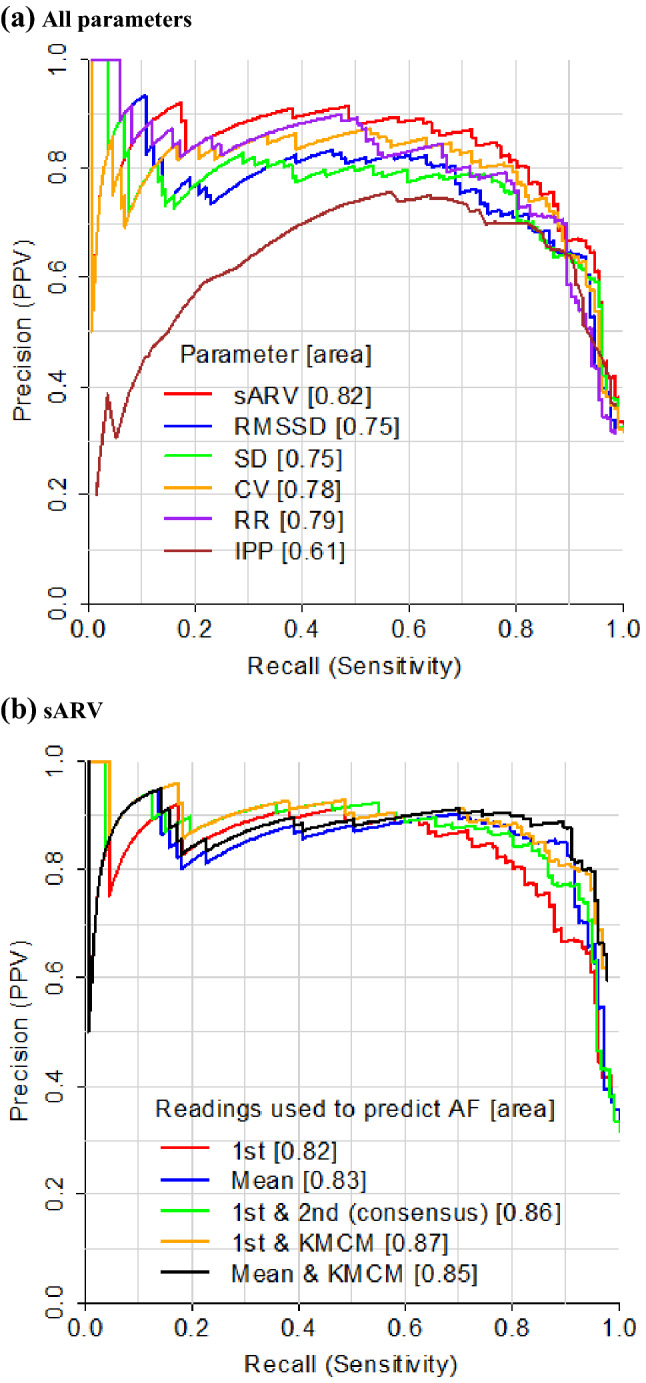
Precision-recall curves for AF detection by pulse rate variability (PRV) with: (**a**) all parameters and, (**b**) sARV. *1st* first reading, *KMCM* Kardia Mobile Cardiac Monitor; other abbreviations are as for Fig. 1. For KMCM curves, AF was indicated when the PRV recording had to exceed the threshold *and* the KMCM output did not read “normal.” Values in brackets give area under each curve and F1 scores are reported in Supplementary Tables 1 and 2.

Next, we varied the PRV threshold for AF detection by PRV and plotted the associated PPV for each corresponding sensitivity value (Fig. 3). When the first PRV recording had to exceed the threshold, PPV and area under the curves were high (Fig. 3a). This was especially for sARV, CV and relative range, which had PPV values of > 0.8 across most sensitivities and the highest areas. Consistent with this, maximum F1 score was highest for these three parameters (Supplementary Table 1). These patters were similar when we based PPV on an AF prevalence of 10% (Supplementary Fig. 3a).

We then focused one of these parameters, sARV, to illustrate the effect of using combinations of measures (Fig. 3b). PPV was higher (by up to 0.18) when the mean of the two sARV recordings had to exceed the threshold (above sensitivities > 0.6) and when both recordings (not just the first one) had to. This was accompanied by increases in maximum F1 score (by up to 0.04) and area under curves (Supplementary Table 2). A similar improvement was observed when the first sARV recording had to exceed the threshold and the KMCM output did not read “normal”. In comparison, PPV at a given sensitivity was higher (by up to 0.21) when using the mean of two sARV readings in combination with a KMCM reading that did not give a “normal” classification. Basing PPV on an AF prevalence of 10% yielded similar patterns (Supplementary Fig. 3b). The abovementioned improvements were observed between sensitivities of 0.65–0.9, corresponding to sARV values of 6–14% (23% of sample).When we classified sARV predictions based on confidence of predictions, combining measurements for “uncertain” predictions only significantly improved PPV (Supplementary Fig. 4).

## Discussion

This diagnostic study of 421 primary care patients showed that AF determined from a 12-lead ECG can be detected with PRV using a BP+ device or with a KMCM. Discrimination and precision were higher in people without a non-AF arrhythmia. The utility of PRV was reinforced by its yield of positive net benefit across a range of threshold probabilities (or clinical preferences to balance false-positives and false-negatives). Finally, using combinations of measurements—two PRV ones or both a PRV and a KMCM recording—increased precision compared with when a single measurement was employed.

### BP+ findings

The accuracy of the PRV parameters (percentage of classifications that were correct) across cut-points reached over 90% (Supplementary Tables 1–3), which is in the range of values observed for this metric in previous AF diagnostic studies (nearly all on outpatient samples) of other BP monitors^4^. Further, the 95% confidence intervals for our AUC values encompassed the AUC values reported in a hospital-based study of the BP+ device^15^. Our work extends these prior findings as we evaluated this device in a different setting (primary care) using different analytical approaches: in combination with the KMCM and using both decision and precision-recall curve analyses. Additional novelty is derived from our use of parameters not assessed (sARV, SD and relative range) in these past studies^4^ or examined each only in one prior study (RMSSD^15^ and IPP^24^).

Our findings are consistent with past studies reporting that, when more than one PRV reading from a BP monitor indicating AF is required for AF diagnosis, discrimination performance improves^22,29^. We add to these past findings by showing that this not only improves PPV at a given sensitivity, but so too does the mean of two PRV measures. Further, the improvement in screening performance when we excluded (from our analysis) non-AF arrhythmias is consistent with prior research on BP monitors which reports that these arrhythmias are present in false-positive groups^22,23,30^ and specificity increases when they are absent^15,22^. This should not deter AF screening in patients with known non-AF arrhythmias as this would lead to missed AF diagnoses and, in cases of a false-positive AF diagnosis, patients may nevertheless benefit from an ECG for any non-AF abnormalities.

### KMCM findings

Compared to KMCM sensitivity and specificity values reported in past diagnostic studies^9–11^, the sensitivity we reported when we excluded unclassified and blank recordings from analyses was similar (97%). But the associated specificity (89%) was lower. If our study had a higher prevalence of non-AF arrhythmias in those without AF, this could explain the specificity difference as when these cases were excluded from analyses, specificity increased (by 0.05; Table 3). This is a novel finding and suggests that, when screening in patients with non-AF arrhythmias, one should be wary of a higher false-positive rate.

Further, sensitivity and specificity were substantially lower when we included unclassified and blank recordings (83% and 68%, respectively), which reflects the fact that our unclassified and blank outputs made up a sizeable proportion (21%) of all KMCM readings. Such recordings increase the risk of missed or delayed AF diagnoses if KMCM measurement is performed by those who cannot interpret ECGs at all or with sufficient accuracy. This problem is highlighted by our finding that these readings were responsible for 86% of all undetected AF cases. To address this, it has been suggested that cardiologists can interpret these readings^13^; but this requires consideration of their busy workflows. However, in people with unclassified or blank KMCM recordings, PRV was able to detect AF accurately, with an AUC of up to 0.89. Thus, using the BP+ alongside KMCM would help to improve AF diagnosis, while minimising cardiologist time commitment.

### Strengths and limitations

A strength of the present study is that, for our BP+ results, we evaluated sensitivity, specificity and PPV at multiple cut-points, which allowed us to determine optimal thresholds for AF detection for our study population; not done in past studies using other devices^4^. Second, we studied people who are most likely to benefit from opportunistic screening: primary care patients, including those without a prior AF diagnosis or at high risk of developing AF^19^ and stroke (elevated CHADS_2_ and CHA_2_DS_2_-VASc scores). As for limitations, studying primary care patients only may restrict our ability to extrapolate findings more widely. A second limitation is that not all measurements were carried out simultaneously with the 12-lead ECG, raising the possibility that AF may have not been consistently present or absent during the ECG and screening measures. Such inconsistency may add to false positives and false negatives, leading to an underestimation of diagnostic accuracy; but we expect the likelihood of this to be low given that all measurements were performed in a narrow time-interval (typically within a few minutes). Third, although the cardiologist who interpreted our ECGs had > 30 years of cardiology experience, involving additional experienced cardiologists in our ECG diagnoses may have provided some benefit.

## Conclusion

In primary care patients, AF can be detected by PRV accurately and by KMCM, especially when used in combination and in the absence of non-AF arrhythmias. Performing two PRV measurements reduces misclassification. Through early AF detection and subsequent treatment, implementing such measurements should have a meaningful impact on the adverse health consequences of AF. For example, a population-based study showed that elevated RMSSD and CV (indicative of AF) predicted increased risk of cerebrovascular events, even in people without known AF or cerebrovascular disease^18^. Intervention studies would clarify whether performing these measurements in clinical practice lead to the expected improvements in AF-related health outcomes.

## Supplementary Information

Supplementary Information.

## Data Availability

No additional data are available. However, the original data that support the findings derived from this study can be requested by emailing the corresponding author.
