# Supplementary material for: Atrial fibrillation detection in primary care during blood pressure measurements and using a smartphone cardiac monitor
Source: Sci Rep. 2021 Sep 6;11:17721. doi: 10.1038/s41598-021-97475-1 (PMC8421380; doi:10.1038/s41598-021-97475-1)
Supplement: Supplementary file 1 — Supplementary Information. [file 41598_2021_97475_MOESM1_ESM.docx]

**Supplementary data**

**Atrial fibrillation detection in primary care during blood pressure measurements and using a smartphone cardiac monitor**

John D. Sluyter, Robert Scragg, Malakai 'Ofanoa, Ralph A. H. Stewart

**Abbreviations used in the Online Supplement:**

1^st^: first pulse rate variability reading; AF: atrial fibrillation; AUC: area under curve; AUPRC: area under precision-recall curve; CV: coefficient of variation; ECG: electrocardiogram; IPP: irregular pulse percentage; KMCM: Kardia Mobile Cardiac Monitor; NPV: negative predictive value; PPV: positive predictive value; RMSSD: root mean square of successive differences; RR: relative range; sARV: standardised average real variability; SD: standard deviation.

**Contents**

**Supplementary Table 1 3**

Diagnostic and predictive values of pulse rate variability parameters with the 1st reading

**Supplementary Table 2 4**

Diagnostic and predictive values of pulse rate variability parameters with the mean of 2 readings

**Supplementary Table 3 5**

Diagnostic and predictive values pulse rate variability parameters with the 1st reading among those without non-AF arrhythmias

**Supplementary Figure 1 6**

Contour plots of area under curve (AUC) for AF detection with 6 pulse rate variability parameters calculated after excluding beats (plots a-e) or classifying them as irregular (plot F) according to beat length (% shorter and longer than mean beat duration)

**Supplementary Figure 2 9**

Examples of suprasystolic brachial blood pressure waveforms with pulse rate variability that is: A) low and, B) high. Left-sided graphs show waveforms in temporal order, delineated by starts of beats (red dotted lines). Right-sided graphs show waveforms superimposed upon one another

**Supplementary Figure 3 10**

Precision-recall curves for AF detection, at an AF prevalence of 10% with: A) all pulse rate variability parameters and, B) sARV. For KMCM curves, AF was indicated when the sARV recording had to exceed the threshold *and* the KMCM output did not read “normal.” Values in brackets give area under each curve

**Supplementary Figure 4 11**

Precision-threshold curves for AF detection by sARV with AF prevalence equal to the: a) sample prevalence and, b) 10%. sARV predictions were classified as “AF unlikely” (sARV<2%; 96% sensitivity), “AF uncertain” (sARV = 2% to threshold) and “AF likely” (sARV>threshold). Combinations of measurements were applied to the “AF uncertain” range across different thresholds. For KMCM curves, AF was indicated when the BP+ recording was within the “AF uncertain” range (2% to threshold) *and* the KMCM output did not read “normal”

**Supplementary Table 1 (1st reading)**

| Parameter | Cut-point ^a^ | Sensitivity | Specificity | PPV | NPV | Accuracy ^b^ | F1 score ^b^ | AUPRC |
| --- | --- | --- | --- | --- | --- | --- | --- | --- |
| sARV (%) | 2 | 0.96 | 0.46 | 0.48 | 0.97 | 0.63 | 0.64 | 0.82 |
|  | 4 | 0.95 | 0.63 | 0.74 | 0.97 | 0.81 | 0.83 |  |
|  | 6 | 0.89 | 0.68 | 0.81 | 0.94 | 0.84 | 0.85 |  |
|  | 8 | 0.87 | 0.76 | 0.87 | 0.94 | 0.87 | 0.87 |  |
|  | 10 | 0.82 | 0.78 | 0.90 | 0.92 | 0.87 | 0.86 |  |
|  | 12 | 0.78 | 0.82 | 0.92 | 0.90 | 0.88 | 0.84 |  |
|  | 14 | 0.63 | 0.88 | 0.96 | 0.85 | 0.86 | 0.76 |  |
|  | 16 | 0.50 | 0.88 | 0.97 | 0.81 | 0.82 | 0.66 |  |
|  | 18 | 0.37 | 0.91 | 0.98 | 0.77 | 0.79 | 0.53 |  |
|  | 20 | 0.23 | 0.86 | 0.98 | 0.74 | 0.75 | 0.37 |  |
| RMSSD (ms) | 20 | 0.95 | 0.43 | 0.43 | 0.95 | 0.59 | 0.59 | 0.75 |
|  | 40 | 0.94 | 0.59 | 0.70 | 0.96 | 0.78 | 0.80 |  |
|  | 60 | 0.86 | 0.69 | 0.82 | 0.93 | 0.83 | 0.84 |  |
|  | 80 | 0.80 | 0.71 | 0.85 | 0.90 | 0.84 | 0.82 |  |
|  | 100 | 0.72 | 0.76 | 0.90 | 0.87 | 0.84 | 0.80 |  |
|  | 120 | 0.63 | 0.82 | 0.94 | 0.85 | 0.84 | 0.75 |  |
|  | 140 | 0.50 | 0.81 | 0.95 | 0.81 | 0.81 | 0.65 |  |
|  | 160 | 0.41 | 0.82 | 0.96 | 0.78 | 0.79 | 0.58 |  |
|  | 180 | 0.31 | 0.79 | 0.96 | 0.75 | 0.76 | 0.47 |  |
|  | 200 | 0.22 | 0.74 | 0.97 | 0.73 | 0.73 | 0.36 |  |
| SD (ms) | 20 | 0.96 | 0.48 | 0.53 | 0.97 | 0.66 | 0.68 | 0.75 |
|  | 40 | 0.90 | 0.64 | 0.77 | 0.94 | 0.81 | 0.83 |  |
|  | 60 | 0.82 | 0.70 | 0.84 | 0.91 | 0.84 | 0.83 |  |
|  | 80 | 0.76 | 0.78 | 0.90 | 0.89 | 0.86 | 0.82 |  |
|  | 100 | 0.60 | 0.79 | 0.93 | 0.83 | 0.82 | 0.72 |  |
|  | 120 | 0.43 | 0.79 | 0.95 | 0.78 | 0.79 | 0.59 |  |
|  | 140 | 0.34 | 0.82 | 0.97 | 0.76 | 0.77 | 0.50 |  |
|  | 160 | 0.16 | 0.75 | 0.98 | 0.72 | 0.72 | 0.27 |  |
|  | 180 | 0.11 | 0.78 | 0.99 | 0.71 | 0.71 | 0.19 |  |
|  | 200 | 0.08 | 0.77 | 0.99 | 0.70 | 0.70 | 0.14 |  |
| CV (%) | 2 | 0.95 | 0.46 | 0.48 | 0.96 | 0.63 | 0.64 | 0.78 |
|  | 4 | 0.92 | 0.64 | 0.76 | 0.95 | 0.81 | 0.83 |  |
|  | 6 | 0.88 | 0.72 | 0.84 | 0.94 | 0.85 | 0.86 |  |
|  | 8 | 0.80 | 0.78 | 0.90 | 0.91 | 0.87 | 0.85 |  |
|  | 10 | 0.70 | 0.84 | 0.94 | 0.87 | 0.86 | 0.80 |  |
|  | 12 | 0.53 | 0.86 | 0.96 | 0.82 | 0.83 | 0.69 |  |
|  | 14 | 0.33 | 0.86 | 0.98 | 0.76 | 0.77 | 0.49 |  |
|  | 16 | 0.15 | 0.83 | 0.99 | 0.71 | 0.72 | 0.25 |  |
|  | 18 | 0.05 | 0.75 | 0.99 | 0.69 | 0.69 | 0.09 |  |
| RR (%) | 5 | 0.96 | 0.42 | 0.39 | 0.95 | 0.57 | 0.55 | 0.79 |
|  | 10 | 0.91 | 0.56 | 0.66 | 0.94 | 0.74 | 0.77 |  |
|  | 15 | 0.90 | 0.64 | 0.77 | 0.94 | 0.81 | 0.83 |  |
|  | 20 | 0.90 | 0.7 | 0.82 | 0.94 | 0.85 | 0.86 |  |
|  | 25 | 0.82 | 0.73 | 0.86 | 0.91 | 0.85 | 0.84 |  |
|  | 30 | 0.80 | 0.77 | 0.89 | 0.90 | 0.86 | 0.84 |  |
|  | 35 | 0.69 | 0.80 | 0.92 | 0.87 | 0.85 | 0.79 |  |
|  | 40 | 0.59 | 0.83 | 0.94 | 0.83 | 0.83 | 0.72 |  |
|  | 45 | 0.45 | 0.90 | 0.98 | 0.79 | 0.81 | 0.62 |  |
|  | 50 | 0.25 | 0.85 | 0.98 | 0.74 | 0.75 | 0.40 |  |
| IPP (%) | 10 | 0.93 | 0.52 | 0.60 | 0.95 | 0.70 | 0.73 | 0.61 |
|  | 20 | 0.88 | 0.65 | 0.78 | 0.93 | 0.81 | 0.83 |  |
|  | 30 | 0.77 | 0.70 | 0.85 | 0.89 | 0.82 | 0.81 |  |
|  | 40 | 0.64 | 0.75 | 0.90 | 0.84 | 0.82 | 0.75 |  |
|  | 50 | 0.47 | 0.73 | 0.92 | 0.79 | 0.78 | 0.63 |  |
|  | 60 | 0.32 | 0.65 | 0.92 | 0.74 | 0.73 | 0.47 |  |
|  | 70 | 0.18 | 0.55 | 0.93 | 0.71 | 0.69 | 0.30 |  |
|  | 80 | 0.08 | 0.39 | 0.94 | 0.69 | 0.67 | 0.14 |  |

^a^ Units are specified in left-hand column. ^b^ Maximum values are shaded.

**Supplementary Table 2 (mean of 2 readings)**

| Parameter | Cut-point ^a^ | Sensitivity | Specificity | PPV | NPV | Accuracy ^b^ | F1 score ^b^ | AUPRC |
| --- | --- | --- | --- | --- | --- | --- | --- | --- |
| sARV (%) | 2 | 0.97 | 0.43 | 0.41 | 0.97 | 0.58 | 0.57 | 0.83 |
|  | 4 | 0.96 | 0.59 | 0.69 | 0.98 | 0.78 | 0.80 |  |
|  | 6 | 0.94 | 0.69 | 0.81 | 0.97 | 0.85 | 0.87 |  |
|  | 8 | 0.92 | 0.80 | 0.89 | 0.96 | 0.90 | 0.90 |  |
|  | 10 | 0.90 | 0.85 | 0.93 | 0.95 | 0.92 | 0.91 |  |
|  | 12 | 0.80 | 0.87 | 0.94 | 0.91 | 0.90 | 0.86 |  |
|  | 14 | 0.68 | 0.90 | 0.97 | 0.87 | 0.87 | 0.80 |  |
|  | 16 | 0.56 | 0.88 | 0.97 | 0.83 | 0.84 | 0.71 |  |
|  | 18 | 0.36 | 0.87 | 0.98 | 0.77 | 0.78 | 0.53 |  |
|  | 20 | 0.20 | 0.81 | 0.98 | 0.73 | 0.73 | 0.33 |  |
| RMSSD (ms) | 20 | 0.98 | 0.42 | 0.38 | 0.97 | 0.56 | 0.55 | 0.81 |
|  | 40 | 0.97 | 0.57 | 0.67 | 0.98 | 0.76 | 0.79 |  |
|  | 60 | 0.91 | 0.67 | 0.80 | 0.95 | 0.86 | 0.85 |  |
|  | 80 | 0.89 | 0.74 | 0.86 | 0.94 | 0.87 | 0.87 |  |
|  | 100 | 0.77 | 0.78 | 0.90 | 0.90 | 0.86 | 0.87 |  |
|  | 120 | 0.67 | 0.82 | 0.93 | 0.86 | 0.85 | 0.78 |  |
|  | 140 | 0.5 | 0.82 | 0.95 | 0.81 | 0.81 | 0.65 |  |
|  | 160 | 0.37 | 0.86 | 0.97 | 0.77 | 0.78 | 0.54 |  |
|  | 180 | 0.28 | 0.90 | 0.99 | 0.75 | 0.76 | 0.44 |  |
|  | 200 | 0.19 | 0.89 | 0.99 | 0.73 | 0.74 | 0.32 |  |
| SD (ms) | 20 | 0.97 | 0.46 | 0.48 | 0.97 | 0.63 | 0.64 | 0.80 |
|  | 40 | 0.93 | 0.61 | 0.73 | 0.96 | 0.79 | 0.82 |  |
|  | 60 | 0.88 | 0.73 | 0.85 | 0.94 | 0.86 | 0.87 |  |
|  | 80 | 0.82 | 0.80 | 0.91 | 0.92 | 0.88 | 0.86 |  |
|  | 100 | 0.65 | 0.87 | 0.96 | 0.85 | 0.86 | 0.77 |  |
|  | 120 | 0.46 | 0.87 | 0.97 | 0.80 | 0.81 | 0.62 |  |
|  | 140 | 0.31 | 0.85 | 0.98 | 0.75 | 0.77 | 0.47 |  |
|  | 160 | 0.19 | 0.81 | 0.98 | 0.72 | 0.73 | 0.32 |  |
|  | 180 | 0.12 | 0.89 | 0.99 | 0.71 | 0.72 | 0.21 |  |
|  | 200 | 0.05 | 0.88 | 1.00 | 0.70 | 0.70 | 0.10 |  |
| CV (%) | 2 | 0.97 | 0.42 | 0.38 | 0.96 | 0.56 | 0.55 | 0.85 |
|  | 4 | 0.94 | 0.61 | 0.72 | 0.96 | 0.79 | 0.82 |  |
|  | 6 | 0.93 | 0.74 | 0.85 | 0.96 | 0.87 | 0.89 |  |
|  | 8 | 0.87 | 0.82 | 0.91 | 0.94 | 0.90 | 0.89 |  |
|  | 10 | 0.78 | 0.90 | 0.96 | 0.91 | 0.90 | 0.86 |  |
|  | 12 | 0.54 | 0.89 | 0.97 | 0.82 | 0.83 | 0.69 |  |
|  | 14 | 0.28 | 0.90 | 0.99 | 0.75 | 0.76 | 0.43 |  |
|  | 16 | 0.12 | 0.89 | 0.99 | 0.71 | 0.72 | 0.21 |  |
|  | 18 | 0.04 | 1.00 | 1.00 | 0.69 | 0.70 | 0.07 |  |
| RR (%) | 5 | 0.96 | 0.42 | 0.39 | 0.95 | 0.57 | 0.55 | 0.88 |
|  | 10 | 0.91 | 0.56 | 0.66 | 0.94 | 0.74 | 0.77 |  |
|  | 15 | 0.90 | 0.64 | 0.77 | 0.94 | 0.81 | 0.83 |  |
|  | 20 | 0.90 | 0.7 | 0.82 | 0.94 | 0.85 | 0.86 |  |
|  | 25 | 0.82 | 0.73 | 0.86 | 0.91 | 0.85 | 0.84 |  |
|  | 30 | 0.80 | 0.77 | 0.89 | 0.90 | 0.86 | 0.84 |  |
|  | 35 | 0.69 | 0.80 | 0.92 | 0.87 | 0.85 | 0.79 |  |
|  | 40 | 0.59 | 0.83 | 0.94 | 0.83 | 0.83 | 0.72 |  |
|  | 45 | 0.45 | 0.90 | 0.98 | 0.79 | 0.81 | 0.62 |  |
|  | 50 | 0.18 | 0.96 | 1.00 | 0.73 | 0.74 | 0.30 |  |
| IPP (%) | 10 | 0.94 | 0.48 | 0.52 | 0.95 | 0.65 | 0.67 | 0.66 |
|  | 20 | 0.93 | 0.61 | 0.73 | 0.95 | 0.79 | 0.81 |  |
|  | 30 | 0.83 | 0.68 | 0.82 | 0.91 | 0.82 | 0.82 |  |
|  | 40 | 0.71 | 0.71 | 0.87 | 0.87 | 0.82 | 0.78 |  |
|  | 50 | 0.52 | 0.75 | 0.92 | 0.81 | 0.79 | 0.66 |  |
|  | 60 | 0.34 | 0.75 | 0.95 | 0.76 | 0.76 | 0.50 |  |
|  | 70 | 0.15 | 0.67 | 0.96 | 0.71 | 0.71 | 0.26 |  |
|  | 80 | 0.02 | 0.2 | 0.97 | 0.68 | 0.67 | 0.03 |  |

^a^ Units are specified in left-hand column. ^b^ Maximum values are shaded.

**Supplementary Table 3 (1st reading, no non-AF arrhythmias)**

| Parameter | Cut-point ^a^ | Sensitivity | Specificity | PPV | NPV | Accuracy ^b^ | F1 score ^b^ | AUPRC |
| --- | --- | --- | --- | --- | --- | --- | --- | --- |
| sARV | 2 | 0.99 | 0.47 | 0.51 | 0.99 | 0.66 | 0.67 | 0.88 |
|  | 4 | 0.98 | 0.66 | 0.77 | 0.99 | 0.84 | 0.86 |  |
|  | 6 | 0.93 | 0.72 | 0.84 | 0.96 | 0.87 | 0.88 |  |
|  | 8 | 0.91 | 0.80 | 0.90 | 0.96 | 0.90 | 0.90 |  |
|  | 10 | 0.86 | 0.83 | 0.92 | 0.94 | 0.90 | 0.89 |  |
|  | 12 | 0.80 | 0.86 | 0.94 | 0.91 | 0.90 | 0.87 |  |
|  | 14 | 0.64 | 0.92 | 0.98 | 0.86 | 0.87 | 0.78 |  |
|  | 16 | 0.52 | 0.92 | 0.98 | 0.82 | 0.84 | 0.68 |  |
|  | 18 | 0.37 | 0.95 | 0.99 | 0.78 | 0.80 | 0.53 |  |
|  | 20 | 0.22 | 0.93 | 0.99 | 0.74 | 0.75 | 0.36 |  |
| RMSSD (ms) | 20 | 0.96 | 0.44 | 0.45 | 0.97 | 0.61 | 0.61 | 0.80 |
|  | 40 | 0.96 | 0.62 | 0.74 | 0.98 | 0.81 | 0.84 |  |
|  | 60 | 0.89 | 0.75 | 0.85 | 0.95 | 0.86 | 0.87 |  |
|  | 80 | 0.83 | 0.75 | 0.88 | 0.92 | 0.86 | 0.85 |  |
|  | 100 | 0.75 | 0.80 | 0.92 | 0.89 | 0.87 | 0.82 |  |
|  | 120 | 0.65 | 0.87 | 0.96 | 0.86 | 0.86 | 0.78 |  |
|  | 140 | 0.51 | 0.86 | 0.96 | 0.82 | 0.82 | 0.67 |  |
|  | 160 | 0.41 | 0.85 | 0.97 | 0.79 | 0.80 | 0.58 |  |
|  | 180 | 0.30 | 0.83 | 0.97 | 0.76 | 0.77 | 0.46 |  |
|  | 200 | 0.21 | 0.77 | 0.97 | 0.73 | 0.74 | 0.34 |  |
| SD (ms) | 20 | 0.99 | 0.50 | 0.55 | 0.99 | 0.69 | 0.71 | 0.80 |
|  | 40 | 0.94 | 0.67 | 0.80 | 0.97 | 0.84 | 0.86 |  |
|  | 60 | 0.86 | 0.73 | 0.86 | 0.93 | 0.86 | 0.86 |  |
|  | 80 | 0.79 | 0.82 | 0.92 | 0.91 | 0.88 | 0.85 |  |
|  | 100 | 0.61 | 0.83 | 0.94 | 0.84 | 0.84 | 0.74 |  |
|  | 120 | 0.43 | 0.81 | 0.96 | 0.79 | 0.79 | 0.59 |  |
|  | 140 | 0.33 | 0.84 | 0.97 | 0.77 | 0.77 | 0.49 |  |
|  | 160 | 0.14 | 0.80 | 0.98 | 0.72 | 0.73 | 0.25 |  |
|  | 180 | 0.11 | 0.92 | 1.00 | 0.71 | 0.72 | 0.19 |  |
|  | 200 | 0.07 | 0.89 | 1.00 | 0.72 | 0.71 | 0.13 |  |
| CV (%) | 2 | 0.98 | 0.47 | 0.51 | 0.98 | 0.65 | 0.67 | 0.86 |
|  | 4 | 0.96 | 0.67 | 0.79 | 0.98 | 0.84 | 0.87 |  |
|  | 6 | 0.92 | 0.76 | 0.87 | 0.96 | 0.89 | 0.89 |  |
|  | 8 | 0.84 | 0.84 | 0.93 | 0.93 | 0.90 | 0.88 |  |
|  | 10 | 0.72 | 0.89 | 0.96 | 0.89 | 0.89 | 0.82 |  |
|  | 12 | 0.57 | 0.91 | 0.98 | 0.84 | 0.85 | 0.72 |  |
|  | 14 | 0.34 | 0.93 | 0.99 | 0.77 | 0.79 | 0.50 |  |
|  | 16 | 0.14 | 0.94 | 1.00 | 0.72 | 0.73 | 0.25 |  |
|  | 18 | 0.04 | 0.80 | 1.00 | 0.70 | 0.70 | 0.07 |  |
| RR (%) | 5 | 0.97 | 0.42 | 0.39 | 0.96 | 0.57 | 0.56 | 0.84 |
|  | 10 | 0.94 | 0.58 | 0.69 | 0.96 | 0.77 | 0.80 |  |
|  | 15 | 0.93 | 0.67 | 0.79 | 0.96 | 0.84 | 0.86 |  |
|  | 20 | 0.93 | 0.74 | 0.85 | 0.96 | 0.87 | 0.89 |  |
|  | 25 | 0.85 | 0.78 | 0.89 | 0.93 | 0.88 | 0.87 |  |
|  | 30 | 0.83 | 0.81 | 0.91 | 0.92 | 0.89 | 0.87 |  |
|  | 35 | 0.73 | 0.84 | 0.94 | 0.88 | 0.87 | 0.82 |  |
|  | 40 | 0.61 | 0.86 | 0.96 | 0.84 | 0.85 | 0.74 |  |
|  | 45 | 0.47 | 0.93 | 0.98 | 0.80 | 0.82 | 0.63 |  |
|  | 50 | 0.25 | 0.88 | 0.98 | 0.74 | 0.76 | 0.40 |  |
| IPP (%) | 10 | 0.96 | 0.54 | 0.63 | 0.97 | 0.73 | 0.76 | 0.69 |
|  | 20 | 0.91 | 0.69 | 0.82 | 0.95 | 0.85 | 0.86 |  |
|  | 30 | 0.78 | 0.76 | 0.89 | 0.90 | 0.86 | 0.83 |  |
|  | 40 | 0.64 | 0.82 | 0.94 | 0.85 | 0.84 | 0.76 |  |
|  | 50 | 0.46 | 0.79 | 0.94 | 0.79 | 0.79 | 0.61 |  |
|  | 60 | 0.31 | 0.71 | 0.94 | 0.75 | 0.75 | 0.46 |  |
|  | 70 | 0.17 | 0.59 | 0.95 | 0.72 | 0.70 | 0.28 |  |
|  | 80 | 0.06 | 0.44 | 0.96 | 0.69 | 0.68 | 0.11 |  |

^a^ Units are specified in left-hand column. ^b^ Maximum values are shaded.

**Supplementary Figure 1**

**A) sARV**

**B) RMSSD**

**Supplementary Figure 1 (continued)**

**C) Standard deviation**

**D) Coefficient of variation**

**Supplementary Figure 1 (continued)**

**E) Relative range**

**F) Irregular pulse period**

**Supplementary Figure 2**

**A) Low pulse rate variability** (sARV=1%, RMSSD=6 ms, SD=4 ms CV=0.5%, RR=1%, IPP=8%)

12-lead ECG cardiologist diagnosis: Sinus rhythm

**B) High pulse rate variability** (sARV=28%, RMSSD=266 ms, SD=141 ms, CV=17%, RR=48 %, IPP=67%)

12-lead ECG cardiologist diagnosis: Atrial fibrillation

**Supplementary Figure 3**

**A) All parameters**

**B) sARV**

**Supplementary Figure 4**

**A) AF prevalence = sample prevalence**

PPV for 1 sARV reading = 0.46

**B) AF prevalence = 10%**

PPV for 1 sARV reading = 0.17
